# An Attempt to Discover Some of the Laws Which Govern Animal Torpidity and Hibernation

**Published:** 1848-03

**Authors:** 


Art. V.—*An Attempt to discover some of the Laws which govern Animal Torpidity and Hibernation.* By Peter A. Browne, LL. D., Member of the National Institute for the Promotion of Science, of the American Association for the Promotion of Science, of the Academy of Natural Science of Philadelphia, of the Academy of Natural Science of Chester County, and Professor of Geology in La Fayette College. Read before the Association of American Geologists and Naturalists, at the eighth Annual Meeting, in Boston, in September, 1847.

The works of the Supreme Architect, being stamped with the seal of his infinite perfections, cannot be exhausted by human industry —*Spallanzani.*

We are very liable to error by drawing hasty conclusions from a few isolated facts, *as they occur in the higher grade of animals,* without availing ourselves of the phenomena of *more extensive animal existence.—Harlan.*

Philadelphia: Barrett & Jones, 34, Carter's Alley. 1847. pp. 32.

The author of this interesting tract defines Torpidity to be "A natural, temporary, intermediate state, between life and death; into which some animals sink, owing to an excess of heat, or of cold, or of drought, or want of oxygen, or for the purpose of enabling them to effect some natural change in their form, such as developing some organ, or of making some addition to their structure;—during which state, (when it is profound), their respiration and digestion are entirely suspended, their circulation reduced to an oscillation of the heart and its immediate vessels, (without, however, destroying the liquidity of their blood), all sensibility and power of voluntary motion are destroyed, and the irritability of their nervous system is greatly diminished."

Of the family of vertebrated animals two classes are hibernating—the mammalia and reptiles. Among the first, are the bat, the hedgehog, bears, raccoons, the marmot, the prairie-dog, the wood-chuck, the water-rat, the field-mouse, porcupines, and some others, belonging to the class of mammals;—and of the cold-blooded vertebrata, all the classes contain hibernators. Of molluscous animals only one order is said to hibernate; namely, the gasteropoda; of the five families of articulated animals, three are hibernating; and of the radiated family, only the rotifers are known to hibernate.

What is the condition of the respiratory function in torpid animals? This is the first point to which our author addresses his inquiries, and he adduces much authority to show that where the torpidity is complete, there is no respiration. Mr. Saissy found that while the hibernating animals examined by him consumed, in the ordinary way, a quantity of oxygen considerable enough for their size, they consumed none at all when entirely torpid. Respiration does not appear to cease all at once, but gradually, in proportion to the degree of torpidity, from the most partial to the most profound; and, according to Saissy, as well as other physiologists, a debasement of temperature corresponds exactly with the diminished consumption of oxygen. The conclusions of our author are thus stated:

"1st. That (as before stated) these animals, when they are enjoying their usual activity, consume a quantity of oxygen considerable enough for their volume.

"2d. That this consumption diminishes in proportion to the diminution of their temperature.

"3d. That they possess at all times the faculty of existing a considerable time in the air that is fit for neither combustion nor respiration, without being much fatigued.

"4th. That during their torpidity, but while respiration is still visible, the consumption of oxygen is very small compared with that of their ordinary state, and

"5th. That during *profound torpidity* there is no consumption of oxygen at all and consequently no *respiration.*"

Dr. Browne adds to the facts derived from other writers on the subject, a curious observation made by Dr. Dickerson relative to the alligator. This animal, according to Dr. D., "hibernates with a pine or cypress knot in his mouth, completely closing the passage of the stomach." The account is, that from the middle of September to the first of October the creature becomes careless of his food; about the latter end of October he selects a pine or cypress knot, with which he stops his mouth, the end extending into his throat. He then crawls into a hole under water, where he remains torpid till the dog-wood blooms in the spring, when he reappears and ejects this piece of wood, which by that time is rounded and smoothed.

The condition of the circulation in torpid animals is the point next examined. All writers on torpidity agree that the blood circulates with less activity in proportion to the depth of the lethargy. A hamster in its natural state has one hundred and fifty pulsations in a minute, and only fifteen when the animal becomes torpid. Toads and frogs with thirty pulsations in the normal condition, have only twelve in their hibernaculum. Saissy even avers that in the hibernating condition the circulation is entirely suspended. Flourens is of the same opinion; and from experiments made by our author on animals of the various classes, he comes to the conclusion arrived at b these eminent naturalists.

The next subject is the digestion of torpid animals. This function, as might be inferred, is entirely null during hibernation.

"Dr. Monroe kept a hedgehog in a room at Edinburgh, without a fire, from November until March. About December, it was affected with an unusual degree of drowsiness, but it continued to eat, sparingly, until the 25th day of that month, when it sank into a state of profound lethargy. It so remained until the 8th of March following, except when occasionally roused, but it would soon return to the torpid state again. During all this time it ate nothing, drank nothing, although food was constantly kept before it.

"So M. White, of Sethbourne, kept a land tortoise forty years. During the summer months it was very voracious, but it gradually became less so towards autumn, and by the middle of November it sank into a torpid state.

"There is no food of which the alligator is so fond as a dog, yet when the time of their hibernation is approaching, they evince an entire indifference to it.—*Dr. Dickerson.*

"M. Saissy also informs us, that the marmot will refuse to eat, and become torpid, even while surrounded by apples, pears, and nuts; and the hedgehog with flesh near him.

"Nature appears to have kindly deprived these animals of the desire for food, foreseeing that they were about to lose the power to digest it.

"Hunter introduced food in the stomachs of lizards about to hibernate, and upon opening them, at different periods of the state, always found the aliment *unaltered.* With some, after the season of hibernation had passed, the food was ejected *unchanged*, (indigested)."

No proof is necessary to establish the fact, that torpid animals are insensible to every external stimulus; but there is one function, the suspension of which has not been fully demonstrated—that of reproduction. Thus Cuvier speaking of the bear, says, that it is in a state of hibernation, more or less profound, "that the female has her young;" and Audibon and Buckman relate the same of the wood-chuck. The torpor of these animals, we conclude, is not very "profound" while this process is going forward.

The anatomical structure and physiology of torpid animals is investigated, but without any very satisfactory results. By some, the thymus gland is believed to bear a decided relation to the torpidity of animals, but the testimony on this point is contradictory. The fat is generally believed to increase before hibernation, and to diminish during the continuance of this state; but here also the experience of observers wants uniformity. Spallanzani found some dormice, caught for his experiments, lean, and others fat; and, on the other hand, Cornish found, by actual experiments, that dormice and bats, by a hibernation of two weeks, lost from five to seven grains of weight. Saissy examined the fat of certain hibernating animals. and found that it was soft and unctuous, like that of a hog; and that it was inodorous, and had an insipid taste. The only effect of torpidity upon it, according to him, was to render it a little more consistent. Our author, notwithstanding, is of the belief, that the fat of hibernating animals "*has* to do with their torpidity." If respiration is suspended in hibernation, we do not see why there should be any consumption of fat, or any loss of weight in the animal; but until that function ceases entirely, the oxygen imbibed will reappear in the shape of carbonic acid formed at the expence of the organism. As the temperature declines in proportion as the breathing becomes feeble, and rises as this process is restored to activity, so the consumption of fat will vary with the state of the respiratory function.

The topic next examined is the causes of torpidity. They are, according to Dr. Browne, immediate and mediate. The immediate causes are cold, heat, drought, want of oxygen, and a natural necessity to repose, while some change takes place in the organization. This last is called metamorphosis.

Cold produces in the human species an insuperable disposition to sleep, as has been exhibited on many memorable occasions. Cold is connected with the torpidity of most hibernating animals, but not with that of all, for some hibernate in hot weather, and some in drought; in some torpidity results from a want of oxygen, and in some attends upon the metamorphosis of the animal.

It is known to all visitors of the Mammoth Cave, that vast numbers of bats congregate in one of the recesses of this cavern to pass through their hibernation. When we were there near the close of September, in the year 1846, the temperature of the outer air was 85°, and yet we found a number of bats in the cave already torpid. The interior of this cave has a perennial temperature of about 56°. What was it that took these animals into their winter quarters while the temperature of the air was still that of high summer? What is it that brings them out in the spring again? What influence reaches them in their deep retreat, to call into activity their slumbering functions? We confess we have no answer.

The *mediate causes* of animal torpidity have been conjectured, without being satisfactorily made out; and hibernation continues to be no less a mystery now than it was when man first speculated on its nature. This tract embraces many curious facts connected with the phenomenon, but affords no adequate solution of the cause.

